# The effect of polymorphisms (174G> C and 572C> G) on the Interleukin-6 gene in coronary artery disease: a systematic review and meta-analysis

**DOI:** 10.1186/s41021-021-00172-8

**Published:** 2021-01-12

**Authors:** Nader Salari, Kamran Mansouri, Amin Hosseinian-Far, Hooman Ghasemi, Masoud Mohammadi, Rostam Jalali, Aliakbar Vaisi-Raygani

**Affiliations:** 1 Department of Biostatistics, School of Health, Kermanshah University of Medical Sciences, Kermanshah, Iran; 2 Medical Biology Research Center, Kermanshah University of Medical Sciences, Kermanshah, Iran; 3 Department of Business Systems & Operations, University of Northampton, Northampton, UK; 4 Student Research Committee, Kermanshah University of Medical Sciences, Kermanshah, Iran; 5 Department of Nursing, School of Nursing and Midwifery, Kermanshah University of Medical Sciences, Kermanshah, Iran

**Keywords:** Coronary artery disease, Polymorphism, Cardiovascular disease, IL-6174 G> C, IL-6 -572C> G, Atherosclerosis

## Abstract

**Background:**

Coronary Artery Disease (CAD) is caused by the blockage of the coronary arteries. it is argued that there has an association between the Interleukin-6 gene and the occurrence of atherosclerosis, coronary artery disease, Due to the short half-life and high variability of Interleukin-6 (IL-6), limited studies have been performed on the association of serum levels of interleukin-6 with coronary artery disease. The aim of this study is to investigate the relationship between IL-6 gene polymorphisms and coronary artery disease.

**Methods:**

This study was conducted as a meta-analysis of selected articles with no lower time limit and upto March 2020. Articles related to the subject were obtained by searching several data sources,such as the SID, IranDoc, Scopus, Embase, Web of Science (ISI), PubMed, Science Direct, and Google Scholar databases. The heterogeneity of the studies was assessed using the I^2^ index in the Comprehensive Meta-Analysis software.

**Results:**

The GG genotype of the IL-6174 G> C polymorphism with a 0.8 odds ratio tended to reduce the risk of CAD by 20%. The odds ratio of CAD in CG and GG genotypes were found to be 1.16 and 1.48 times respectively, indicating the increasing effect of these two genotypes. In the IL-6-572 C>G polymorphism, CG and GG genotypes increased the risk of CAD by 1.21 and 1.27 times respectively, and the CC genotype tended to reduce the risk of CAD by 15%, considering the odds ratio of 0.85.

**Conclusion:**

This study showed a relationship between IL-6174G> C and Interleukin-6 (IL-6) 572 C>G genes and coronary artery disease. Moreover, the protective effects of GG genotype in IL-6 gene 174 G> C and CC genotype in IL-6 gene 572 C>G gene were reported. The study also confirmed that the CG and CC genotypes of the G>C IL-6174 gene have an increasing effect on coronary artery disease. Moreover, CG and GG genotypes in the IL-6 gene 572 C>G increased the risk of developing CAD. It should be noted that the increased risk of developing CAD was limited to meta-analytic studies in reported literatures.

## Background

Cardiovascular diseases are known to be a leading disorder that embrace a wide range of specific diseases such as vascular disorders, Myocardial Infarction (MI), and congenital heart disease. In fact, such diseases involve disorders of the cardiovascular and blood circulation systems. Cardiovascular diseases are known to be a top cause of human mortality worldwide. Studies have shown that cardiovascular disorders are more likely to cause death in the general population than other diseases such as infectious diseases, maternal and infant diseases, nutritional disorders, and cancers [1–4]. The mortality rate from cardiovascular diseases is reported to be 45% [5]. The constant accumulation of fat, immune cells, and fibrous tissues in the lining of the arteries can block the arteries. This can lead to decreased alertness, decreased blood flow, and eventually a cardiovascular disease [6].

Atherosclerosis is a cardiovascular disorder and a chronic inflammatory response by the body that has stable and unstable periods. This disorder occurs in the large and medium arteries. The cause of this disease is fat and cholesterol accumulation in the wall of the arteries [7, 8]. Atherosclerotic lesions are caused by asymmetric thickening of the inner layer of the arteries [9]. Acute Coronary artery Syndrome (ACS), Coronary Artery Disease (CAD), Myocardial Infarction (MI), stable and unstable angina, stroke, transient ischemic attack and peripheral arterial disease are known as atherosclerotic subunits [10].

Coronary Artery Disease (CAD) is caused by a blockage of the coronary arteries. The disease can cause myocardial ischemia and eventually necrosis of the heart. CAD is considered the leading cause of death in different populations. The formation of atherosclerotic plaques is a major factor in the advancement of CAD. Although the the mechanism is not unknow and the research of that is limited, the role of inflammation in the occurrence of atherosclerotic plaques cannot be ignored [11]. Angina and myocardial infarction can be considered as some of the clinical indicators of this disorder [11–13]. Several types of immune cells, including macrophages,and T and B lymphocytes also accumulate in the arterial walls and are involved in the development of atherosclerotic disorders through cytokines and other mediators [14].

Cytokines are small proteins that, in addition to the immune system, have a wide range of physiological roles. These proteins are produced by different types of cells. Functional pleiotropy is one of the characteristics of this group of materials and makes cytokines able to play a regulatory role in cell differentiation and proliferation activities according to cell type and status. Different types of cytokines include interleukins, interferon, and growth factors [15, 16].

There are several factors that contribute to coronary artery disease. However, these factors vary from one person to another. Genetics is considered to be one of the factors influencing the development of CAD. Some studies have reported 50 risk points in the human genome that can influence CAD development [17]. Extensive genomic studies demonstrate that genetic factors increase the chances of developing CAD by 1.1 to 1.3 times [18]. It has also been shown that hereditary factors account for 30–60% of interpersonal differences in CAD [19]. The severity of the genetic effect depends on factors such as the age of the person at the onset point and also the type of disease [4].

The Interleukin-6 (IL-6) encoding gene is located on the 7P21 chromosome and contains 6 exons. Most studies have focused on the Interleukin-6 (IL-6) gene promoter region. This is because many single-nucleotide polymorphisms, such as IL-6-1744 G / C polymorphisms, IL-6 -572 C / G, and IL-6 -598, are located in this region [20]. Sie et al. (2006) argue that IL-6174 polymorphism is effective in transcribing the Interleukin-6 (IL-6) gene and, consequently, in the plasma level of IL-6 [21]. On the other hand, it is argued that there has been an association between the IL-6572 gene and the occurrence of atherosclerosis, coronary artery disease, and MI [22].

IL-6 is the main stimulus for the acute phase response and stimulates liver synthesis and enhances the C-reactive protein (CRP) [23]. This protein is a precursor to vascular atheromatobiotic disease and is involved in the pathogenesis of atherosclerosis. Elevated plasma levels of IL-6 and CRP increase the risk of death in the elderly. On the other hand, serum IL-6 levels are high in people with unstable angina, which is a symptom of coronary artery disease [23–25].

**Fig. 1:**
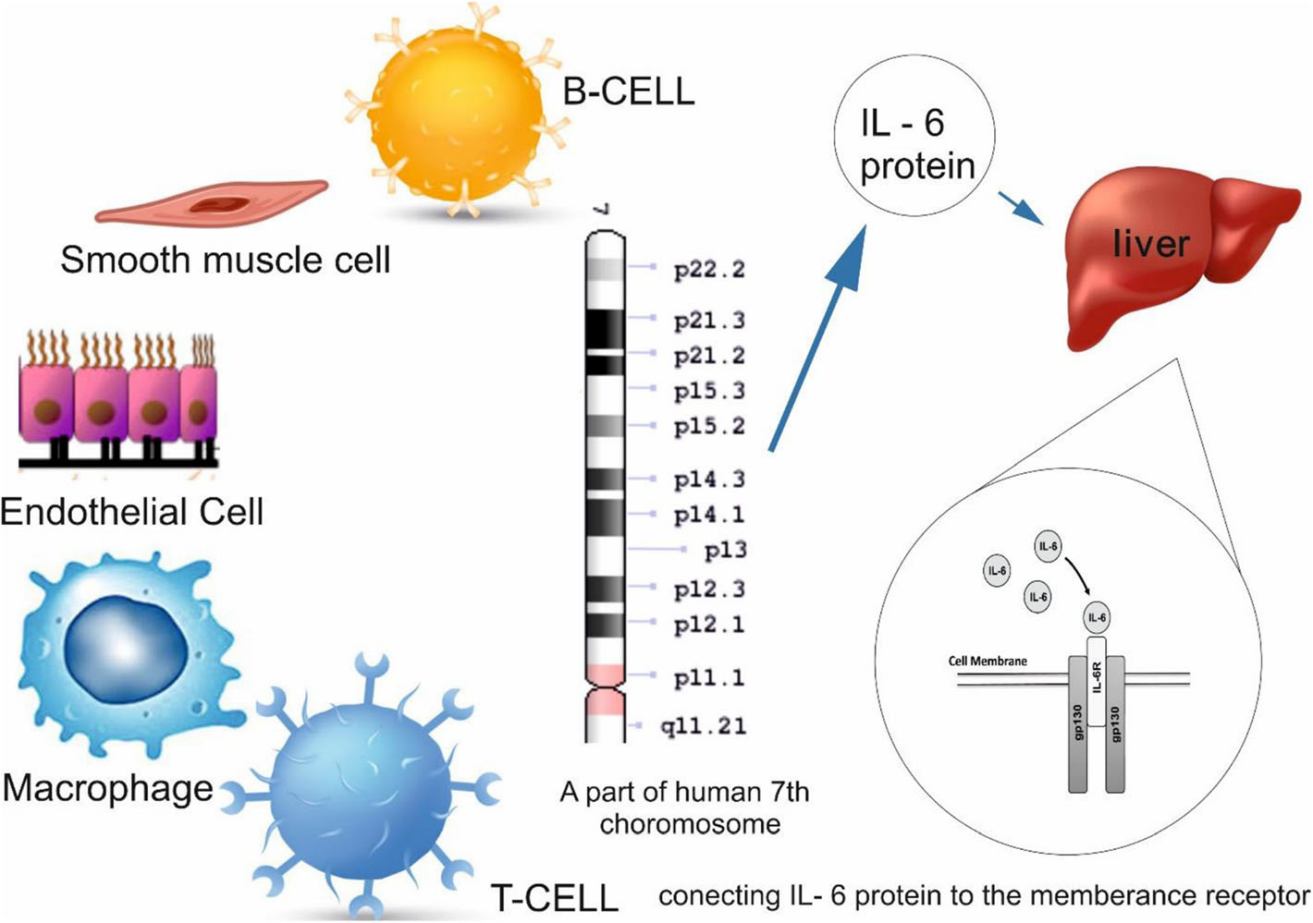
Immune cells, smooth muscle cells, and endothelial cells synthesize interleukin-6 from human 7th chromosome. In the liver, interleukin 6 binds to the IL-6 membrane receptor, activating gp130 and stimulating the classical signaling pathway

IL-6 is known as a cytotoxic polytropic. This protein is involved in activities such as activating macrophages, stimulating B and T cells, and thymocytes in the process of differentiation, regulating restorative activities, and replicating and inducing the activities of the immune system, and the cardiovascular and nervous systems. Interleukin-6 family of proteins include Leukemia Inhibitory Factor (LIF), cardiothropin-1 (CT-1), IL-6, and IL-11. Interleukin-6 is expressed by tissue immune cells. Cardiovascular cells such as endothelial cells, smooth muscle cells, and ischemic myocytes also play a part in the expression of IL-6 proteins [15, 16, 23, 26] (Fig. 1).

IL-6 connects to its membrane receptor; This receptor consists of two sub-units IL-6 and gpl30. Once IL-6 is connected to IL-6R, it activates soluble IL-6R. The IL-6_SIL-6R complex as a potent agonist activates Glycoprotein 130 (a signal transmission component), and this process enhances the IL-6 mechanism. IL-6 plays an important role as a mediator of inflammation.

Due to the short half-life and high variability of Interleukin-6 (IL-6), limited studies have been performed on the association of serum levels of interleukin-6 with coronary artery disease [27]. Moreover, the interactions between cardiovascular risk factors, inflammatory mechanism and atheromatosis are complex [25]. Given the importance of recognizing people prone to heart disease, the risks associated with the disease and since the disease has a high mortality rate, Knowing the factors that affect its occurrence can be effective in macro-planning to control and prevent the occurrence of the disease and its complications in different populations. In this study, the relationship between IL-6 gene polymorphism and coronary artery disease is examined.

## Methods

### Search method

This study was performed to determine the relationship between cytokine gene polymorphism and cardiovascular disorders by systematic review and meta-analysis. To collect data in this study, Iranian and international databases of Embase, Scopus, Web of Science, PubMed, Science Direct, ProQuest, Google Scholar, SID, and Irandoc were searched. Search process in the above-mentioned databases were conducted using the keywords: (“Interleukin gene” OR “ Interleukin-6 (IL-6) gene” OR “IL-6 polymorphism” OR “IL-174G / C OR 572C / G OR 597G / A OR IL-6R OR RS1800795 OR RS1800796 OR RS1800797 OR “cytokine gene“) AND (“cardiovascular diseases“ OR CVD OR CHD OR CAD OR OR MI OR atherosclerosis OR “heart failure” OR “heart diseases“ myocery “OR“ coronary art infarction “OR“ angina pectoris “OR“ ischemic heart “). These keywords and their possible combination were searched in international databases, and for Persian databases the search was done using the Persian equivalent of the keywords expressed. Moreover, searches in Google Scholar were conducted separately both in English and in Persian, in order to identify gray literature. Assessment of relevant webpages, as well as analysing the references within collected sources were also conducted.

### Selection criteria, and article assessment

After the search process, all the articles were collected in the EndNote software,and the duplicate articles were deleted. Criteria for entering articles into the study were: 1- case control studies and 2- cohort research, and 3- studies that link the genes expressing cytokines and cardiovascular disorders; Criteria for excluding the studies were: 1- cross-sectional studies 2- case reports 3- interventional studies 4-letters to editor 5- studies where their full text were not available, and 6- studies in which the study population had an underlying disease.

Then, a list of article titles and abstracts was put together, and after redacting the details of the articles, their full texts were provided to the reviewers. Each article was independently reviewed by two reviewers, and any disagreement was then reviewed by a third reviewer.

**Fig. 2:**
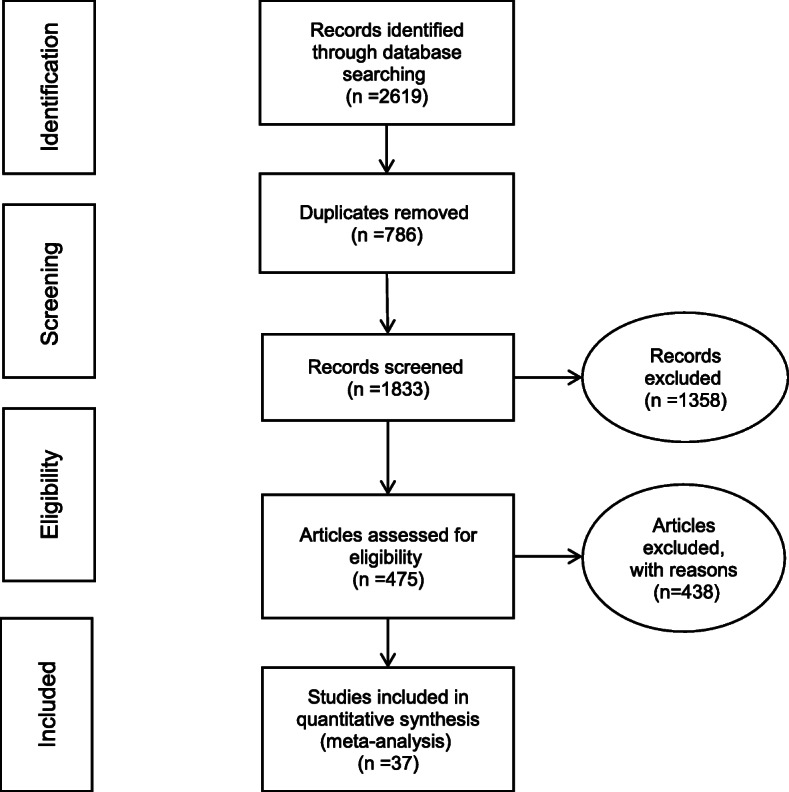
PRISMA flow diagram illustrating the four step process for identifuing teh articles for meta-analysis

For assessing the quality of the collected articles, STROBE checklist was used. This checklist includes 22 criteria, 18 of which are used publicly for all observational studies and 4 items are used exclusively based on the type of study. The checklist criteria include study objectives, determining the appropriate sample size, type of study, sampling method, research community, data collection method, defining variables and how to review the samples, study data collection tools, study objectives, the statistical test used, and the results of the study. The maximum score for articles could be 31, and articles with a score below 14 were excluded from the study. The studies were then meta-analysed using the four step of (i.e. identification of articles, screening, review of admission criteria, and finally articles submitted to meta-analysis) PRISMA 2009 process (Fig. 2).

### Statistical analysis

In this study, the heterogeneity of studies was examined using the I^2^ test. In general, heterogeneity is classified into three categories, heterogeneity less than 25% (low heterogeneity), between 25 to 75% (medium heterogeneity) and above 75% (high heterogeneity). Data were meta-analysed using the Comprehensive Meta-analysis software (Biostat, Englewood, NJ, USA version 3).

## Results

This study examined the relationship between IL-6572G> C and IL-6174 C> G gene polymorphism and coronary artery disease following a systematic review and meta-analysis. After searching various databases, 2619 articles were collected. 317 articles from EMBASE, 399 articles from ProQuest, 345 articles from PubMed, 149 articles from Science Direct, 313 articles from Scopus, 395 articles from Web of Science, 6 articles from SID, 10 articles from Irandoc. and 685 articles from Google Scholar. 786 duplicate articles were removed, and subsequently 1358 other articles were deleted after reviewing their title and abstract. 475 articles were then entered the secondary evaluation phase. By examining the full text of the articles in terms of relevance and quality, another 438 articles were excluded. and finally 37 articles entered the meta-analysis stage (Tables 1 and 2) [28–61].

**Table 1:** Characteristics of studies that entered the meta-analysis stage

Rows	Name [references]	country	Year of publish	Mean age of case group (SD)	Mean age of control group (SD)	Case Group Population	Control group population
1	Amorim, Fernanda Gobbi [28]	Brazil	2011	54.9 + 1.1	54.9 + 1.1	72	71
2	Ansari, Wafa Munir [29]	Pakistan	2017	42±3.80	42±3.80	340	310
3	Babu, Baddela M. V. Srikanth [30]	India	2012	53.56 ±11.72	52.62 ± 8.45	651	432
4	Banerjee, Indranil [31]	India	2009	56.3 ± 12.1	56.0 ± 9.5	210	232
5	Basso, F [32].	America	2002	56±5	56±5	498	1108
6	Bhanushali, A. A [33].	India	2013	48±11	50±11	100	150
7	Chen, H [34].	China	2018	61.00 ± 10.49	60.37 ± 10.38	322	429
8	Elsaid, A [35].	Egypt	2014	53.54 ± 9.1	45.3 ± 7.2	108	143
9	Fan, W. H [36].	China	2011	52.1 ± 6.8	52.3 ± 8.8	84	130
10	Fragoso, J. M [22].	Mexico	2010	58.88 ±11.7	56.03 ±4.13	284	247
11	Galimudi, R. K [37].	India	2014	65±5	64±6	200	200
12	Gao, Y. L. [38]	China	2016	61.50±10.53	52.54±10.65	275	286
13	Ghazouani, Lakhdar [39]	Tunisia	2010	58.1±12.0	56.7±14.1	418	406
14	Humphries, S. E [40].	England	2001	56·7±3·6	56·0±3·4	162	2589
15	Indumathi, B [41].	India	2019	56.00 ± 12.00	–	265	205
16	Jabir, N. R [42]	Saudi Arabia	2017	60.6±8.85	47.7±5.06	90	89
17	Jia, X. W [43].	China	2010	–	–	231	210
18	Jun, M [44].	China	2017	63.5±13.6	64.2±14.1	860	862
19	Li, L. [45]	China	2015	–	–	365	365
20	Maitra, A [46].	India	2008	57.17 ± 9.22	45.53 ± 5.98	46	40
21	Mao, L. [47]	China	2016	62.65 ± 9.72	56.82 ± 9.80	224	260
22	Mastana, S [48].	India	2017	49.51±11.06	45.02±14.46	143	136
23	Mitrokhin, V [49].	Russia	2017	70.37±13.45	74.94±7.43	198	116
24	Nauck, M [50].	Germany	2002	63.77±9.89	58.30±11.83	2575	729
25	Omer, W [51].	Turkey	2016	42 ±3.80	39 ± 7.8	340	310
26	Phulukdaree, Alisa [52]	India	2013	37.5	–	41	61
27	Rios, D. L. S [53].	Brazil	2010	–	–	414	253
28	Sarecka-Hujar, Beata [54]	Poland	2008	43.8±6.1	35.4±10.4	177	202
29	Satti, H. S [55].	Pakistan	2013	46.4 ± 18.7	35.2 ± 17.4	36	52
30	Sekuri, C [56].	Turkey	2007	46.3 ±7.8	44.3 ±7.2	115	105
31	Shabana, N. A [4].	Pakistan	2018	59.10 ± 12.64	56.0 ± 10.4	404	219
32	Sun, G. Q [57].	China	2014	61.2 ± 8.5	56.4 ± 11.6	296	327
33	Tong, Z. C [58].	China	2013	61.4±8.7	60.6±9.6	326	341
34	Tütün, Ufuk [59]	Turkey	2006	29.3± 4.3	31.1 ± 4.0	21	50
35	Wang, K [60]	China	2015	65.4 ± 8.4	64.9 ± 8.2	402	402
36	Yang, H. T [61].	China	2015	–	–	410	410
37	Yao, H. M [17].	China	2016	62.64±8.43	61.43±7.85	275	296

**Table 2:** Odds ration of studies that entered the meta-analysis stage (IL-6 -174 G>C and coronary and IL-6572 C>G and coronary artery disease)

IL-6 -174 G>C and coronary artery disease	Name	GG case group	GG control group	OR	CG case group	CG control group	OR	CC case group	CC control group	OR
	Amorim, Fernanda Gobbi	29	41	0.493 (0.254–0.961)	37	27	1.723 (0.885–3.352)	6	3	2.061 (0.495–8.582)
	Ansari, Wafa Munir	242	236	0.774 (0.545–1.100)	85	71	1.122 (0.782–1.610)	13	3	4.068 (1.148–14.414)
	Babu, Baddela M. V. Srikanth	134	135	0.570 (0.432–0.753)	294	206	0.903 (0.708–1.153)	123	91	0.873 (0.645–1.182)
	Banerjee, Indranil	159	171	1.112 (0.723–1.710)	43	57	0.791 (0.505–1.239)	8	4	2.257 (0.670–7.609)
	Basso, F.	161	375	0.934 (0.75–1.170)	259	549	1.103 (0.893–1.363)	78	185	0.927 (0.694–1.237)
	Bhanushali, A. A.	77	121	0.802 (0.433–1.488)	20	25	1.250 (0.652–2.398)	3	4	1.129 (0.247–5.155)
	Chen, H.	155	190	1.168 (0.874–1.560)	218	133	4.665 (3.420–6.364)	56	27	3.135 (1.931–5.089)
	Elsaid, A.	23	55	0.433 (0.290–0.647)	55	49	1.991 (1.194–3.320)	26	0	92.188 (5.545–1532.625)
	Fan, W. H.	84	129	1.958 (0.048–48.619)	0	1	0.511 (0.021–12.668)	–	–	–
	Galimudi, R. K.	72	113	0.433 (0.245–0.766)	102	69	1.976 (1.322–2.955)	26	18	1.511 (0.800–2.854)
	Gao, Y. L.	183	224	0.551 (0.378–0.802)	50	47	1.130 (0.729–1.751)	42	15	3.257 (1.761–6.024)
	Ghazouani, Lakhdar	298	297	0.911 (0.672–1.237)	110	102	1.064 (0.779–1.455)	10	7	1.397 (0.527–3.706)
	Humphries, S. E.	40	827	0.699 (0.484–1.008)	95	1263	1.489 (1.079–2.054)	25	470	0823 (0.531–1.275)
	Indumathi, B.	163	145	0.661 (0.448–0.976)	99	57	1.549 (1.044–2.296)	3	3	0.771 (0.154–3.860)
	Jabir, N. R	62	63	0.914 (0.483–1.731)	25	23	1.104 (0.569–2.139)	3	3	0989 (0.194–5.034)
	Jun, M.	450	503	0.783 (0.648–0.948)	338	311	1.147 (0.944–1.34)	72	48	1.549 (1.062–2.262)
	Li, L.	213	245	0.686 (0.508–0.928)	113	105	1.110 (0.809–1.525)	39	15	2.791 (1.510–5.160)
	Maitra, A.	36	30	1.2 (0.441–3.267)	10	7	1.310 (0.447–3.838)	0	3	0.115 (0.006–2.301)
	Mao, L.	142	193	0.601 (0.408–0.887)	45	45	1.201 (0.760–1.899)	37	22	2.140 (1.221–3.752)
	Mastana, S.	105	91	1.366 (0.816–2.287)	32	39	0.717 (0.417–1.232)	1	1	0.951 (0.059–15.352)
	Mitrokhin, V.	62	32	1.197 (0.722–1.985)	100	58	1.020 (0.645–1.614)	36	26	0.769 (0.437–1.355)
	Nauck, M.	838	230	1.047 (0.877–1.249)	1238	355	0.976 (0.828–1.150)	499	144	0.976 (0.794–1.201)
	Omer, W.	13	3	4.068 (1.148–14.414)	85	71	1.122 (0.782–1.610)	242	236	0.774 (0.545–1.1)
	Phulukdaree, Alisa	29	34	1.919 (0.827–4.451)	11	19	0.811 (0.337–1.950)	1	8	0.166 (0.020–1.378)
	Rios, D. L. S.	254	151	1.072 (0.779–1.476)	126	86	0.850 (0.608–1.168)	34	13	1.652 (0.854–3.194)
		43	60	0.759 (0.481–1.2)	92	105	1 (0.668–1.497)	42	37	1.387 (0.844–2.281)
	Satti, H. S.	18	38	0.368 (0.150–0.902)	11	14	1.194 (0.468–3.049)	7	0	26.695 (1.472–484.196)
	Sekuri, C.	61	57	0.951 (0.560–1.617)	49	41	1.159 (0.676–1.966)	5	7	0636 (0.185–2.824)
	Shabana, N. A.	194	96	1.184 (0.850–1.647)	133	90	0.703 (0.501–0.988)	99	33	1.830 (1.185–2.824)
	Sun, G. Q.	191	236	0.701 (0.500–0.985)	61	63	1.088 (0.734–1.612)	44	28	1.865 (1.128–3.082)
	Tong, Z. C.	201	220	0.884 (0.646–1.212)	87	98	0.903 (0.643–1.267)	38	23	1.824 (1.061–3.136)
	Tütün, Ufuk	11	35	0.471 (0.165–1.345)	6	15	0.933 (0.303–2.870)	4	0	25.971 (1.330–507.193)
	Wang, K	153	182	0.743 (0.561–0.984)	171	169	1.021 (0.771–1.350)	51	78	1.657 (1.129–2.432)
	Yang, H. T.	198	239	0.668 (0.507–0.880)	163	146	1.193 (0.899–1.583)	49	25	2.090 (1.264–3.456)
	Yao, H. M.	256	282	0.669 (0.329–1.362)	19	14	1.495 (0.734–3.043)	–	–	–
IL-6572 C>G and coronary artery disease	name	CC case group	CC control group	OR	CG case group	CG control group	OR	GG case group	GG control group	OR
	Basso, F.	1	2	1.113 (0.101–12.299)	56	116	1.083 (0.773–1.519)	425	959	0.905 (0.668–1.224)
	Chen, H.	228	176	3.487 (2.563–4.743)	158	131	2.192 (1.624–2.958)	43	33	1.849 (1.146–2.985)
	Fan, W. H.	42	95	0.368 (0.207–0.656)	38	32	2.530 (1.407–4.547)	4	3	2.117 (0.462–9.706)
	Fragoso, J. M.	23	36	0.516 (0.297–0.899)	146	103	1.479 (1.049–2.086)	115	108	0.876 (0.620–1.37)
	Gao, Y. L.	114	129	0.862 (0.617–1.204)	134	135	1.063 (0.763–1.481)	27	22	1.306 (0.725–2.354)
	Humphries, S. E.	0	9	0.836 (0.048–14.423)	19	225	1.396 (0.849–2.296)	135	2224	0.821 (0.535–1.259)
	Jabir, N. R	3	0	7.160 (0.364–140.655)	22	21	1.048 (0.528–2.080)	59	54	1.234 (0.671–2.266)
	Jia, X. W.	79	88	0.721 (0.490–1.060)	130	107	1.239 (0.851–1.803)	22	15	1.368 (0.690–2.714)
	Jun, M.	427	555	0.545 (0.450–0.662)	337	264	1.460 (1.196–1.781)	96	43	2.393 (1.648–3.475)
	Li, L.	132	166	0.679 (0.505–0.914)	165	155	1.118 (0.834–1.498)	68	43	1.715 (1.134–2.592)
	Maitra, A.	8	6	1.193 (0.376–3.787)	15	23	0.358 (0.148–0.862)	23	11	2.636 (1..068–6.505)
	Mao, L.	81	106	0.823 (0.570–1.189)	110	127	1.010 (0.707–1.445)	33	27	1.491 (0.866–2.567)
	Mitrokhin, V.	0	2	0.115 (0.005–2.424)	16	10	0.932 (0.408–2.128)	182	104	1.313 (0.598–2.881)
	Sun, G. Q.	37	39	1.055 (0.653–1.705)	69	73	1.058 (0.727–1.538)	190	215	0.934 (0.672–1.298)
	Tong, Z. C.	179	180	1.089 (0.03–1.477)	110	121	0.926 (0.673–1.274)	37	43	0.887 (0.555–1.417)
	Wang, K	176	192	0.852 (0.645–1.124)	187	181	1.062 (0.805–1.402)	39	29	1.382 (0.837–2.283)

### Gene Interleukin-6 (IL-6) 174G> C

#### Investigation of heterogeneity and publication bias (GG, CG, CC genotypes)

The heterogeneity of the studies was investigated using I^2^ test and based on this test, the heterogeneity in GG, CG and CC genotypes were calculated as I^2^ = 59.1%, I^2^ = 79.2%, and I^2^ = 70% respectively The results demonstrate a high level of heterogeneity among articles, and therefore the random effects model was used to amalgamate the results from these studies. The publication bias was calculated using the Egger’s test; the test did not find bias in any of the genotypes, since the results were not statistically significant (*P*> 0.05) (Table 3). One of the most important reasons for the high heterogeneity in the study is the difference in the volume of case and control samples in the studied studies. The number of samples in each study is adjusted according to the objectives of that study and as reported in Table 1 among all Studies are different.

**Table 3:** Heterogenity and publication bias in the studied genotypes

Genotype	I^2^	Egger’s test
GG	59.1%	0.740
CG	79.2%	0.231
CC	70%	0.056

#### Meta-analysis of genotype GG

**Fig. 3:**
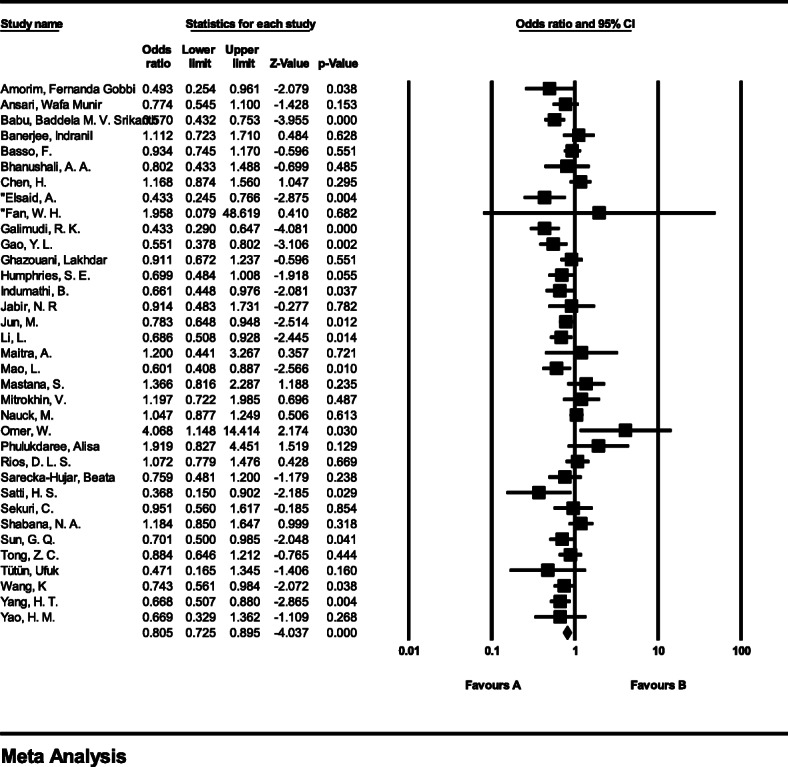
The odds ratio of GG genotype in patients with coronary artery disease

The total number of samples entered in the study was 11,463 and 12,316 for the case and control groups respectively. The odds ratio of GG genotype in patients with coronary artery disease based on meta-analysis of 35 studies was 0.8 (95% CI:0.72–0.89), which tends to reduce the risk of CAD by 0.2, meaning that people with this genotype are 20% less likely than others to tends coronary artery disease (Fig. 3). Figure 3 illustrates the odds ratio based on the model of random effects. The black small rectangle is the odds ratio, and the length of the line on which the square is located, is a representative of the 95% significance level for each study. The diamond sign represents the odds ratio for all studies.

#### Meta-analysis of genotype CG

**Fig. 4:**
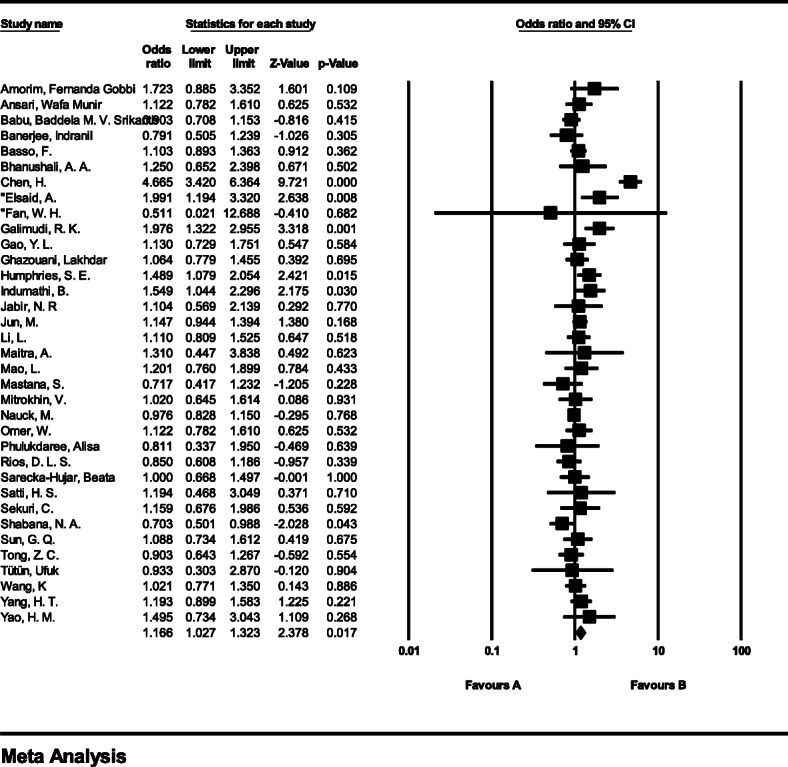
The odds ratio of CG genotype in patients with coronary artery disease

The total number of samples entered in the study was 11,463 and 12,316 for the case and control groups respectively. The odds ratio of CG genotype in patients with coronary artery disease based on meta-analysis of 35 studies by 1.16 (95% CI:1.02–1.32), which indicates the increasing effect of CG genotype with 0.16, meaning that people with this genotype are 16% more likely than others to have coronary artery disease (Fig. 4). Figure 4 illustrates the odds ratio based on the model of random effects. The black small rectangle is the odds ratio, and the length of the line on which the square is located, is a representative of the 95% significance level for each study. The diamond sign represents the odds ratio for all studies.

#### Meta-analysis of genotype CC

**Fig. 5:**
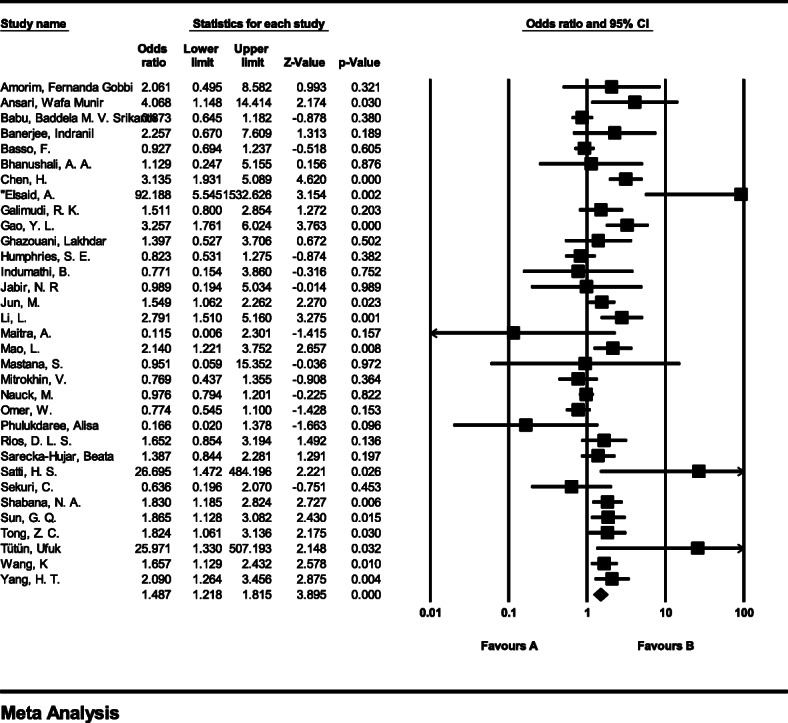
The odds ratio of CC genotype in patients with coronary artery disease

The total number of samples entered in the study was 11,104 and 11,890 for the case and control groups respectively. The odds ratio of CC genotype in patients with coronary artery disease based on meta-analysis of 33 studies was 1.48 (95% CI:1.21–1.91), which indicates the increasing effect of CC genotype by 0.48, meaning that people with this genotype are 48% more likely than others to have coronary artery disease (Fig. 5). Figure 5 illustrates the odds ratio based on the model of random effects. The black small rectangle is the odds ratio, and the length of the line on which the square is located, is a representative of the 95% significance level for each study. The diamond sign represents the odds ratio for all studies.

### Gene Interleukin-6 (IL-6) 572C> G

#### Investigation of heterogeneity and publication bias (GG, CG, CC genotypes)

The heterogeneity of the studies was investigated using I^2^ test and based on this test, the heterogeneity in GG, CG and CC genotypes were calculated as I^2^ = 59.6%, I^2^ = 62.4%, and I^2^ = 87.7% respectively The results demonstrate a high level of heterogeneity among articles, and therefore the random effects model was used to amalgamate the results from these studies. The publication bias was calculated using the Egger’s test; the test did not find bias in any of the genotypes, since the results were not statistically significant (*P*> 0.05) (Table 4).

**Table 4:** Heterogenity and publication bias in the studied genotypes

Genotype	I^2^	Egger’s test
GG	59.6%	0.135
CG	62.4%	0.290
CC	87.7%	0.821

#### Meta-analysis of genotype GG

**Fig. 6:**
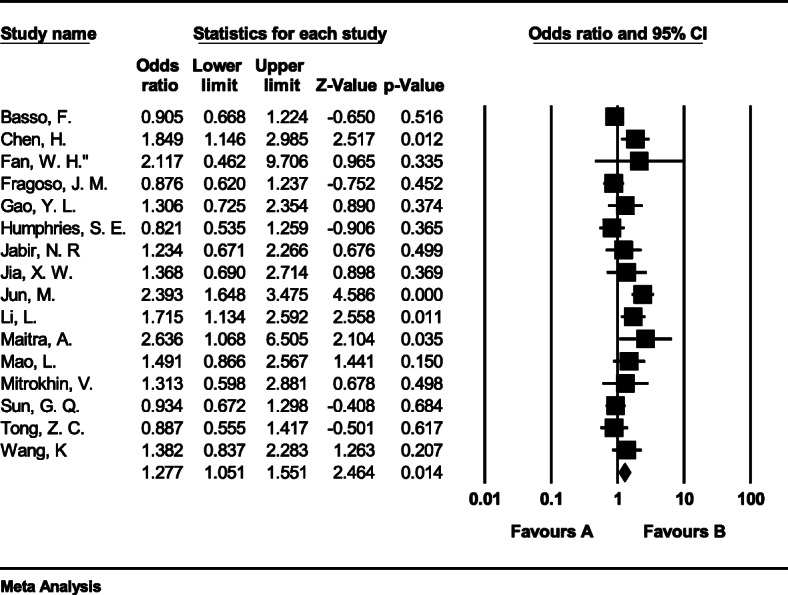
The odds ratio of GG genotype in patients with coronary artery disease

The total number of samples entered in the study was 4663 and 7801 for the case and control groups respectively. The odds ratio of GG genotype in patients with coronary artery disease based on meta-analysis of 16 studies was 1.27 (95% CI:1.05–1.55), which indicates the increasing effect of GG genotype by 0.27, meaning that people with this genotype are 27% more likely than others to have coronary artery disease (Fig. 6). Figure 6 illustrates the odds ratio based on the model of random effects. The black small rectangle is the odds ratio, and the length of the line on which the square is located, is a representative of the 95% significance level for each study. The diamond sign represents the odds ratio for all studies.

#### Meta-analysis of genotype CG

**Fig. 7:**
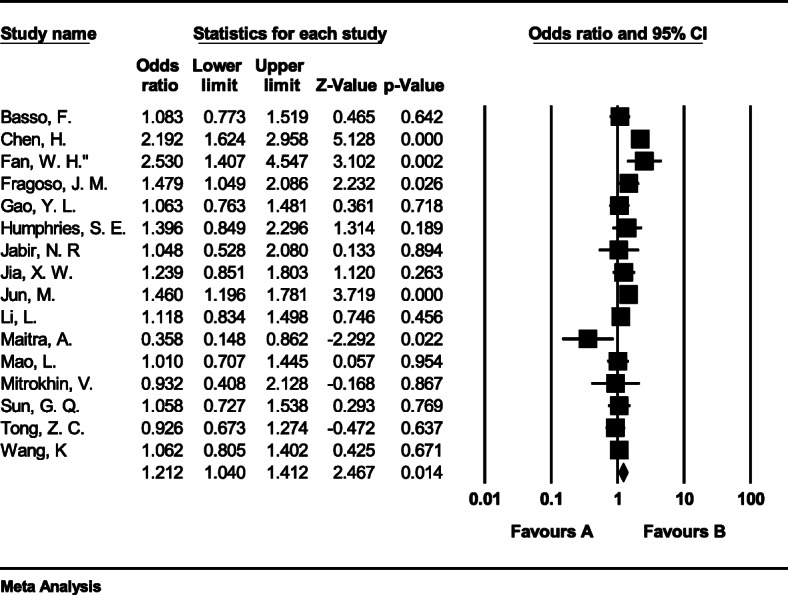
The odds ratio of CG genotype in patients with coronary artery disease

The total number of samples entered in the study was 4663 and 6615 for the case and control groups respectively. The odds ratio of CG genotype in patients with coronary artery disease based on meta-analysis of 16 studies was 1.21 (95% CI:1.04–1.41), which indicates the increasing effect of CG genotype by 0.21, meaning that people with this genotype are 21% more likely than others to have coronary artery disease (Fig. 7). Figure 7 illustrates the odds ratio based on the model of random effects. The black small rectangle is the odds ratio, and the length of the line on which the square is located, is a representative of the 95% significance level for each study. The diamond sign represents the odds ratio for all studies.

#### Meta-analysis of genotype CC

**Fig. 8:**
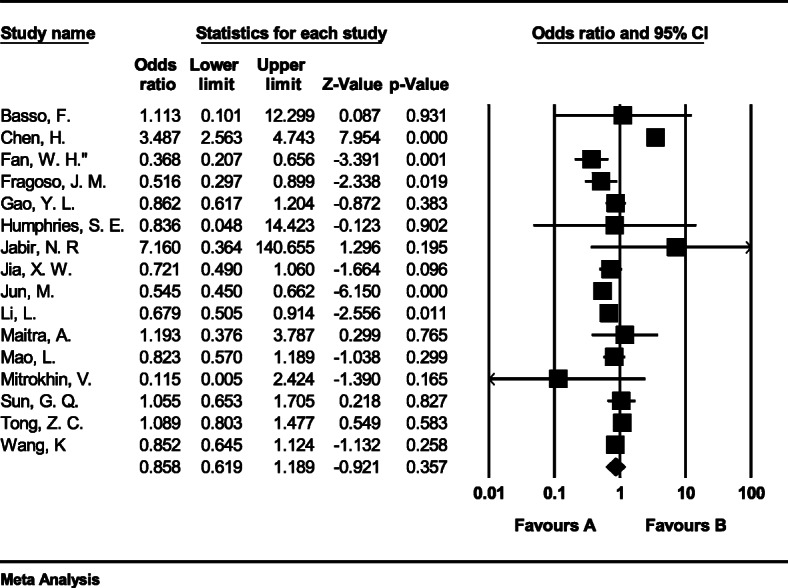
The odds ratio of CC genotype in patients with coronary artery disease

The total number of samples entered in the study was 4165 and 7801 for the case and control groups respectively. The odds ratio of CC genotype in patients with coronary artery disease based on meta-analysis of 16 studies was 0.85 (95% CI:0.61–1.18), which tends to reduce the risk of CAD by 0.15, meaning that people with this genotype are 15% less likely than others to tends coronary artery disease (Fig. 8). Figure 8 illustrates the odds ratio based on the model of random effects. The black small rectangle is the odds ratio, and the length of the line on which the square is located, is a representative of the 95% significance level for each study. The diamond sign represents the odds ratio for all studies.

**Fig. 9:**
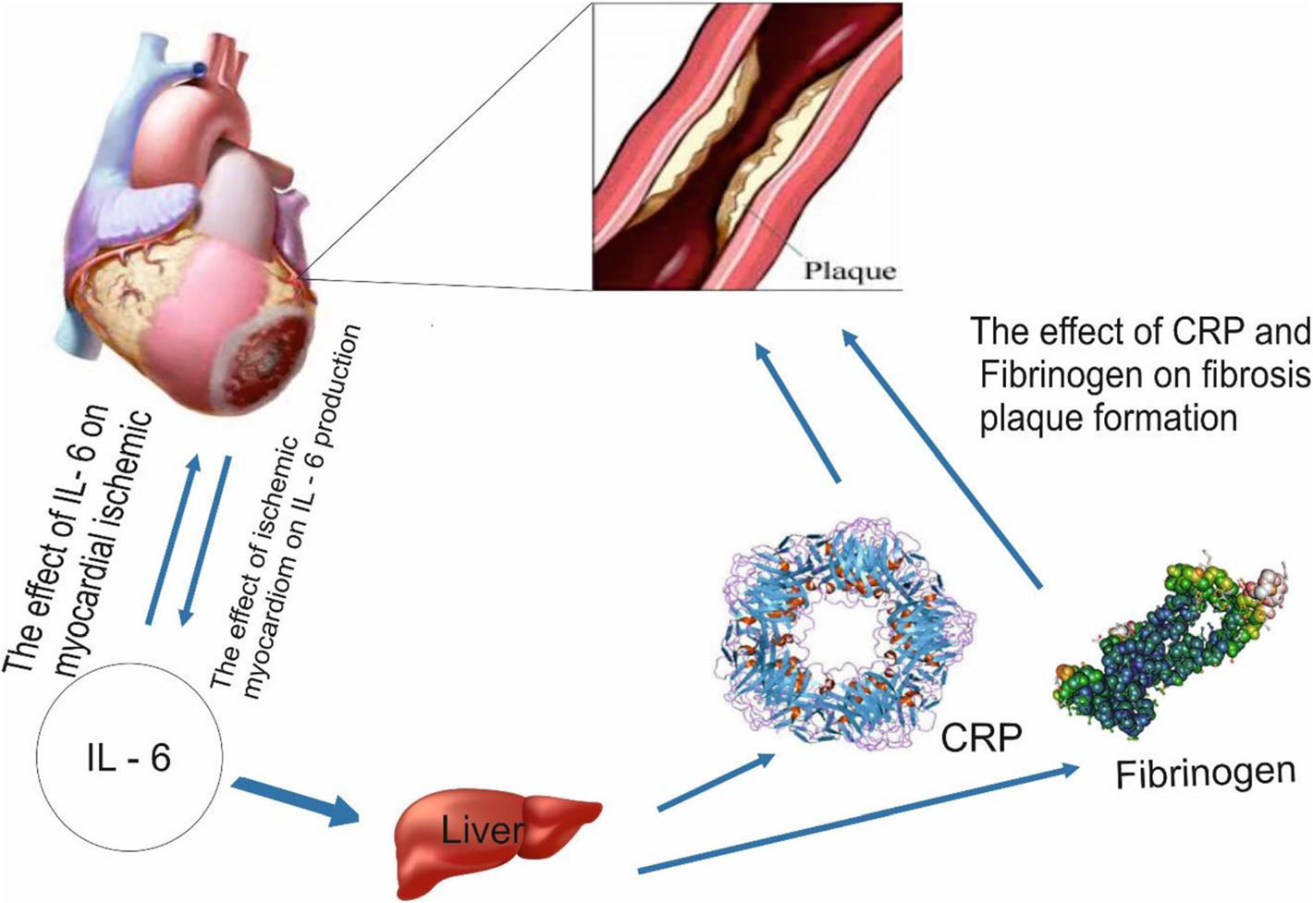
The relationship between IL-6, CRP and cardiovascular disease: Interleukin 6 affects CID and causes CRP and fibrinogen production. These two substances cause the formation of fibrosis plaque in the coronary arteries. Furthermore, the effect of ischemic myocardial infarction on IL-6 production and the role of IL-6 in cardiovascular disease has been shown to be two-sided

## Discussion

Cardiovascular disorders, including coronary artery disease, have a very complex etiology. However, the impact of environmental and genetic factors on the occurance of these diseases has been verified. Factors influencing the incidence of this disease are divided into two categories: modifiable and unmodifiable [62, 63]. In other words, factors such as diabetes, smoking, obesity, blood pressure and stress are among the factors that can be corrected. Genetics, age and gender are considered as unmodifiable factors. Known genetic factors slightly increase the risk of coronary artery disease. However, genetic patterns that greatly increase the risk of CAD are still unknown [38, 64]. However, genetic susceptibility that exposes individuals to abnormal levels of atherosclerosis also intensified the risk of developing CAD [62–65].

The development of techniques in the field of biomolecules has made it possible to conduct comrehensive studies on polymorphisms. Examination of the polymorphism of cytokine genes, including interleukin-6, has shown that the production of these proteins varies from one person to another. It has also been shown that different pathways of Interleukin-6 (IL-6) protein expression have specific biological roles in the development of CAD susceptibility [50].

In this study, the relationship between two different polymorphisms of the Interleukin-6 (IL-6) gene was investigated by a systematic review and meta-analysis. After analyzing data related to Interleukin-6 (IL-6) 174G> C polymorphism and its relationship to CAD, it was highlighted that CC genotype has a more increasing effect (with 48% higher likelihood) than other genotypes. The heterozygous genotype also increases the chances of developing CAD by 16%, whereas the GG genotype offers a 20% protective effect; in other words, the risk of developing CAD in carriers of this genotype is 20% lower than others. It has been observed that in this polymorphism, allele C acts as a risk factor for coronary artery disease. Moreover, by analyzing the information related to Interleukin-6 (IL-6) -572C> G polymorphism, it was found that CC genotype exerts its protective effect by 15% in this polymorphism, whereas CG and GG genotypes increase the risk of developing CAD by 21 and 27% respectively. In this polymorphism, allele G increases the risk of CAD and is considered as a risk factor [50–55].

A study by González-Castro et al. (2019) demonstrated that the Interleukin-6 (IL-6) 174 gene was associated with CAD, highlighting that the homozygous genotype and the heterogeneous genotype increased the risk of developing CAD by up to 50 and 10% respectively. According to this study, dominance will increase the risk of CAD by 23%. This gene also generally increases the risk of cardiovascular disease [66]. Zheng et al., (2012), after reviewing 27 studies with systematic review and meta-analysis, argued that there was no association between Interleukin-6 (IL-6) -174G> C polymorphism and an increased risk of Coronary Heart Disease (CHD) [67]. According to another meta-analysis conducted by Zhang et al. (2018), after reviewing 46 studies related to Interleukin-6 (IL-6) 174 G> C, it was stated that the GG genotype exerts a 19% protective effect. CG and CC genotypes also increase the risk of CAD by 5 and 35% respectively. In this study, the relationship between Interleukin-6 (IL-6) -572 and CAD was also measured. According to the analysis, the GG genotype has a protective effect and the CG and GG genotypes have an increasing effect. Nevertheless, the results of the analysis of CC and GG genotypes in the Interleukin-6 (IL-6) 572 gene in the study are different from the results of our analysis [68]. The study of Aker et al. (2009) also showed that in Interleukin-6 (IL-6)-174 polymorphism, CC genotype increased the risk of CAD more than CG and GG genotypes [69].

According to literature, factors such as inflammation and activation of immune cells play a role in the development of coronary artery syndrome [70]. That is to say, inflammation and atherosclerosis have a similar pathophysiological pathway [65].

It is possible that small changes in serum levels of inflammatory agents may be a factor in the clinical course of the disease [71] Inflammatory cytokines, such as interleukins, are involved in some CAD predisposing factors. These include LDL cholesterol, arterial blood pressure, and diastolic heart function. Existing research works show that high Interleukin-6 (IL-6) concentration is directly related to the development of CAD [72]. According to a study by Toutouzas, et al. (2017) various Interleukin-6 (IL-6) 174 G> C polymorphism genotypes are associated with serum Interleukin-6 (IL-6) levels. Moreover, according to the same research, the CC genotype of this polymorphism increases serum IL-6 levels more than other genotypes. In other words, the C allele in this polymorphism has an increasing effect on the serum level of Interleukin-6 (IL-6), increasing the risk of CAD development, and by affecting the formation of coronary plaques, it increases the clinical symptoms in a multi-year course [71]. Moreover, another research work demonstrated that Interleukin-6 (IL-6) 174 polymorphism had no significant correlation with the serum IL-6 levels [73].

Plasma concentrations of IL-6 can reflect the severity of inflammation caused by fibrous plaques. Therefore, by monitoring the concentration of IL-6 in a person’s bloodstream, the risk of developing CAD can be measured [74]. Studies show that IL-6 is a biomarker with better sensitivity and characteristics than CRP for the diagnosis of cardiovascular disease [75]. A study by Lindmark et al. (2001) argue that IL-6 levels also play a role in mortality from heart disease, in orther words, the higher the plasma levels of this substance, the higher the mortality rate (Fig. 9) [76].

As mentioned earlier, CAD lesions are caused by fibrous plaques surrounded by lipids. Early atherosclerotic plaques are composed of CRP that may be similar to monocytes over time. In other words, the expression of CRP and adhesion molecules can stimulate chemokines and co-activate endothelial cells with lipopolysaccharides. Adhesive molecules are known to mediate T cells that are involved in creating immune responses to inflammation [38, 77].

Existing research works demonstrate that Interleukin-6 (IL-6) gene polymorphisms affect plasma levels of hsCRP and fibrinogen (47). High levels of CRP are known to be a risk factor for development of CAD. Moreover, risks associated with other various factors such as obesity and high blood pressure are seen in these people [65]. One common approach is to measure serum concentrations of anti-inflammatory agents such as hs CRP and interleukin 6. Both substances are considered as a risk factor for development of heart disease, and assessment of their levels are commonly used to determine the risk of inflammation [78]. Klimushina et al. (2019) argued that women carrying the GL Interleukin-6 (IL-6) 174 polymorphism had higher serum hsCRP levels than the CC genotype [73]. A study by Champan et al. (2003) which was conducted in Australia found that CG and CC genotypes increased the probability of developing fibroid plaques in the Australian population by 1.39 and 2.22 times respectively [78].

### Limitations

One of the limitations of this study was the lack of access to the full text of a number of studies; moreover, articles which were in a language other than English or Persian were considered for this systematic review and meta-analysis.

## Conclusions

This study demonstrated that there is an association between IL-6174 and IL-6572 genes and the occurrence of coronary artery disease. According to the analyses conducted in this work, the protective effect of GG genotype in IL-6174 gene and CC genotype in IL-6572 gene were illustrated. It was also stated that the CG and CC genotypes of the IL-6174 gene have an increasing effect on coronary artery disease. Furthermore, it was observed that CG and GG genotypes in the IL-6572 gene increase the probability of developing CAD. And in general, people with the CC genotype of the IL-6174 gene are more at risk of developing CAD than others. In future studies, we can compare the plasma levels of interleukin-6 with each polymorphism and the risk of developing CAD.

## Data Availability

Datasets are available through the corresponding author upon reasonable request.

## Abbreviations

IL-6: Interleukin-6
CAD: Coronary Artery Disease
MI: Myocardial Infarction
ACS: Acute Coronary artery Syndrome
SID: Scientific Information Database
STROBE: Strengthening the Reporting of Observational Studies in Epidemiology
PRISMA: Preferred Reporting Items for Systematic Reviews and Meta-Analysis
